# Plasma mineral status after a six-month intervention providing one egg per day to young Malawian children: a randomized controlled trial

**DOI:** 10.1038/s41598-023-33114-1

**Published:** 2023-04-24

**Authors:** Marina Perez-Plazola, Jenna Diaz, Christine P. Stewart, Charles D. Arnold, Bess L. Caswell, Chessa K. Lutter, E. Rochelle Werner, Kenneth Maleta, Jay Turner, Pradeep Prathibha, Xuan Liu, Emmanuel Gyimah, Lora Iannotti

**Affiliations:** 1 Washington University School of Medicine in St. Louis, St. Louis, USA; 2 Division of Pediatric Gastroenterology, Hepatology and Nutrition, Department of Pediatrics, Washington University School of Medicine in St. Louis, 660 South Euclid Avenue, Campus, Box 8208, St. Louis, MO 63110 USA; 3 Institute for Global Nutrition, Department of Nutrition, University of California Davis, Davis, USA; 4 Western Human Nutrition Research Center, U.S. Department of Agriculture, Agricultural Research Service,, Davis, USA; 5 International Development Group, RTI International US, Triangle Park, USA; 6 School of Global and Public Health, Kamuzu University of Health Sciences, Blantyre, Malawi; 7 Division of Engineering Education Energy, Environmental Energy and Chemical Engineering, Washington University in St. Louis, St. Louis, USA; 8 Institute for Public Health, Washington University in St. Louis, St. Louis, USA

**Keywords:** Diagnostic markers, Biomarkers, Diseases, Health care, Metals, Calcium, Iron

## Abstract

Mineral deficiencies are common in children living in low-resource areas. Eggs are a rich source of essential nutrients and have been shown to improve growth in young children, although little is known about their impact on mineral status. Children aged 6–9 months (n = 660) were randomized to receive either one egg/day for 6-months or no intervention. Anthropometric data, dietary recalls, and venous blood were collected at baseline and 6-months follow-up. Quantification of plasma minerals (n = 387) was done using inductively coupled plasma-mass spectroscopy. Difference-in-difference mean plasma mineral concentrations was determined from baseline and follow-up values and assessed between groups by intention-to-treat using ANCOVA regression models. Prevalence of zinc deficiency was 57.4% at baseline and 60.5% at follow-up. Mean difference (MD) of plasma magnesium, selenium, copper, and zinc levels were not different between groups. Plasma iron concentrations were significantly lower in the intervention compared to the control group (MD = − 9.29; 95% CI: − 15.95, − 2.64). Zinc deficiency was widely prevalent in this population. Mineral deficiencies were not addressed with the egg intervention. Further interventions are needed to improve the mineral status of young children.

## Introduction

Adequate mineral status is necessary for normal cellular function, and deficiencies directly affect childhood growth and development^1–4^. Macro-minerals, such as calcium and magnesium, are found abundantly in the human body and are essential for bone ossification and neuromuscular functioning, respectively^5^. Trace elements are found in smaller quantities in the body but are equally important for many biochemical and physiological processes. Iron and zinc, essential trace elements, are two of the most recognized mineral deficiencies in malnourished children worldwide^3,6^. Iron deficiency anemia results in poor tissue oxygenation, impaired nervous system development, and decreased immunity^6^. Zinc is required for proper function of the immune system and deficiencies increase morbidities from diarrheal and respiratory illnesses^7,8^. Other less recognized trace elements, such as selenium, have a critical role in cognitive development, and immune and thyroid function^9–11^.

Children with malnutrition rarely have deficiencies in only one of these minerals. Multiple deficiencies are prevalent, and have a compounding detrimental effect on children’s health^3^. Young children, especially those under 24 months of age and living in low- and middle-income countries (LMICs), are particularly vulnerable to mineral deficiencies, growth faltering, and infectious diseases^2,12^. Increasing intake of animal source foods (ASFs) and diversifying the diets of young children can help them meet their nutritional needs and avoid long-lasting or even permanent deficits^13–18^. Eggs are affordable, nutrient dense ASFs, and abundant in selenium, providing nearly 77% of a child’s daily selenium requirement with consumption of a single egg^19,20^. Additionally, eggs have been shown to decrease stunting, increase nutrient intake, and improve dietary adequacy in young children^14,21–24^.

The Mazira Project carried out in Malawi sought to replicate and expand on the Lulun Project in Ecuador, in which early introduction of eggs in the complementary feeding process reduced stunting by 47%^14^. To date, findings from the Mazira trial have not shown intervention effects on stunting, anemia, or plasma choline levels^25–27^. Children in the intervention group did have higher intakes of fat, protein, and selenium compared to the control group, but the prevalence of micronutrient inadequacy was high in both groups based on current nutrient intake recommendations for age^24^. Our primary objective was to evaluate the effect of egg intervention on mineral status in the children participating in the Mazira study. We hypothesized those in the intervention group would have higher plasma mineral concentrations compared to the control group at six months, with the greatest effect being on selenium levels due to its high concentration in eggs. Additionally, in an exploratory analysis, we examined whether sex of the child and baseline maternal education would act as moderators in the effect of the intervention on plasma mineral levels.

## Methods

### Study design

The Mazira Project was a randomized controlled trial (clinicaltrials.gov registry NCT03385252; 12/28/2017), conducted in the Lungwena and Malindi areas of Mangochi District in rural Malawi from February 2018 to January 2019. Full details of the study protocol have been previously published^25^. In brief, a total of 660 children aged 6–9 months were randomized to intervention or control group for a six-month period. Participants were recruited from home visits on household listings and community outreach events. General description of the study, purpose, activities, and procedures were discussed with the caregivers. Written informed consent was obtained from each caregiver at baseline prior to data collection. The study was reviewed and approved by ethics committees of the College of Medicine in Malawi, Washington University in St Louis, and the University of California, Davis. The entirety of the research study was performed in accordance with all relevant guidelines and regulations, including the Declaration of Helsinki. This study examining the effect of the intervention on plasma mineral levels, is a secondary analysis of the data collected from the Mazira Project where the primary outcome was linear growth^25^. Thus, our sample size was calculated for the primary trial outcome, linear growth, and not for plasma mineral concentrations.

### Randomization, masking, and intervention

Randomization occurred at the end of a baseline study visit, using a 1:1 allocation ratio in blocks of 10. A member of the study team invited each household caregiver to select and open a sealed, opaque envelope from a basket containing the child’s group, under the supervision of a study-independent community member who was blinded to group assignment. When the number of available envelopes dropped below three, a new block of 10, which included five from the intervention and five from the control group was added to the basket. The intervention was not blinded to the participants or the staff completing the home visits. However, the staff collecting data related to study outcomes—including anthropometrists, interviewers, phlebotomists, and laboratory professionals—were blinded to group allocation.

Families in the intervention group received weekly egg deliveries and were visited twice per week to observe a caregiver feeding the child. Each household in the intervention group was provided with an egg storage basket and directions on food hygiene and handwashing. Suggestions for egg preparation, cooking recipes, and instructions to not share the intervention eggs with other family members were also provided. However, due to the high likelihood of egg-sharing with other household members, families were given an additional seven eggs per week.

Households in the control group were also visited twice per week and given messages about food hygiene and handwashing during food preparation but no eggs or other food items. During the trial, control group households received incentive items such as a bucket, hand washing basin, laundry tub, and lidded plastic bin. At the end of the study, they received a food basket containing items like flour, sugar, oil, and 15 eggs, as well as food storage containers and cloth diapers. The total of these incentive items matched the value of the eggs provided to the intervention households.

### Participants

Infants born out of a singleton pregnancy, aged 6–9 months, residing within the catchment areas of St. Martins Rural Hospital in Malindi and the Lungwena Health Center were eligible to participate in the study. Children with conditions that might affect growth and development, such as, severe anemia (hemoglobin < 5 g/dL), mid-upper arm circumference < 12.5 cm, bipedal oedema, or acute illness or injury warranting hospital referral, were not eligible to participate. Those who were identified as too ill for the study, were referred to the hospital for medical care. History of food allergy or any allergic reactions during egg test feeding at enrollment were excluded from participation. Families who planned to leave the study area within the next six months were also excluded from study participation.

### Data collection

Assessments for growth, development, and dietary intake were obtained from all participating children at baseline and at the six-month follow-up. Anthropometric measures were converted to z-scores per the World Health Organization (WHO) Growth Standards^28^. Household demographic characteristics, including assets and caregiver educational attainment, as well as food insecurity and home environment, were obtained during enrollment and household visits^29,30^. Detailed dietary intake was obtained using a multi-pass 24-h recall at baseline, 3-month and 6-month follow-up^24,31^. Trained nurses and phlebotomists obtained non-fasting venous blood samples at baseline and follow-up clinic visits per WHO Blood Draw guidelines^32^. Samples were collected using a 23-gauge Sarstedt Safety-Multifly® butterfly needle. Hemoglobin (Hgb) concentration was obtained using a portable spectrophotometer (Hemocue Hb 201, HemoCue Inc., Angelholm, Sweden) and malaria using a rapid diagnostic test kit (SD Bioline Malaria Ag P.f/Pan, Abbott Diagnostics, Lake Forest, IL).

Blood was then placed into a trace metal certified, S-Monovette® 5.5-ml plasma collection tube containing lithium heparin. No more than five milliliters were obtained from each child. The tubes were inverted 10 times and then placed on ice. Samples were centrifuged, and plasma was aliquoted into cryovials within one hour of the blood draw. Cryovials were stored in a − 20 °C freezer until the end of the day and then transferred on ice to a local laboratory, where they were placed in a − 80 °C freezer for long-term storage. The plasma aliquots were shipped on dry ice from Malawi to the University of California, Davis by international courier. Further aliquoting at University of California, Davis was performed to conduct various assays^26,27^. One aliquot was shipped on dry ice to the Jay Turner Laboratory at Washington University in St. Louis for mineral analysis.

### Plasma analysis

Analysis to quantify mineral abundances in plasma samples drawn at baseline and 6-months was conducted by the Jay Turner Group in the McKelvey School of Engineering at Washington University. Samples were digested with microwave assistance based on a protocol adapted from Harrington et al., to optimize recovery of iron, selenium, and zinc^33^*.* In brief, 250 µL aliquot of plasma, along with 300 µL concentrated nitric acid (Suprapure®, Millipore Sigma), 200 µL concentrated hydrochloric acid (Fisher Chemical), 100 µL non-stabilized 30% hydrogen peroxide (Thermo Scientific), and 1150 µL ultrahigh water (≥ 18 MΩ/cm resistivity, MilliQ Water Purification System, EMD Millipore) was added to an acid-washed vessel. Then, the samples were sealed and digested (MARS 6, CEM) by heating to 200 °C for seven minutes and held for nine minutes. After cooling, the digestates were spiked with 0.3 mL pure ethanol (Thermo Scientific) and diluted with ultrahigh quality water to a total volume of 15 mL.

Mineral concentrations were measured using inductively coupled plasma mass-spectrometry (ICP-MS; NexION® 2000, Perkin-Elmer), using standard operating conditions (Supplementary Table S1). Minimum detection limits (Supplementary Table S2) were determined using method blanks and instrument performance was validated using standard reference material for trace elements in human serum (Seronorm™, SERO AS). We used ICP-MS to measure Ca isotope 43 as recovery is high (92.5%) and then later extrapolated to total calcium levels.

Standard reference ranges and cutoffs were used to determine prevalence of mineral deficiency (Supplementary Table S3)^34,35^. Prevalence of zinc deficiency was set using previously published guidelines^7^. Copper deficiency was determined using the adult standard, which is < 50 μg/dL, since children after six months of age have more comparable levels to older children and adults than the low levels seen in neonates^34,36^. The cutoff of < 1 mg/dL, was used for hypomagnesemia given symptoms, such as fatigue, weakness, muscle spasms, tremors, and loss of appetite, are almost always present^34,37,38^. The prevalence of deficiency was not determined for iron or selenium since there is no agreed upon standard.

### Statistical analysis

A detailed Statistical Analysis Plan was developed for this analysis and is publicly available at https://osf.io/vfrg7. Descriptive statistics for demographic characteristics were determined for those at enrollment. Since the subsample who consented to provide blood and who had sufficient blood volume for analysis may have differed from the main sample, baseline characteristics were compared across intervention arms using t-tests for continuous variables and chi-squared tests for categorical. The primary outcomes in this analysis are plasma mineral concentrations (magnesium, copper, iron, selenium, and zinc) at the 6-month follow up. This was assessed using a complete-case analysis, where outcome measures were analyzed using ANCOVA regression models to estimate the mean difference between groups, controlling for the baseline measure of the outcome variable. Minimally adjusted analysis included baseline concentrations, time of sample collection, and time of last meal. In the fully adjusted analysis, we additionally controlled for covariates including age, sex, maternal education, socio-economic status, and number of children in the household under five years of age if associated with the outcome (p < 0.1).

Effect modification analysis with the primary outcome of mineral status was used to examine the interaction of maternal education and child sex on the intervention. Significant interactions were considered for *p* < 0.1. Child sex was chosen given biological differences and care practices that may influence the effect of the intervention. Maternal education was also chosen given previous studies showing a significant association (*P* = 0.024) between higher levels of maternal education and increased length-for-age *z* scores in children in the Mazira study^25^.

## Results

### Participant characteristics

**Figure 1:**
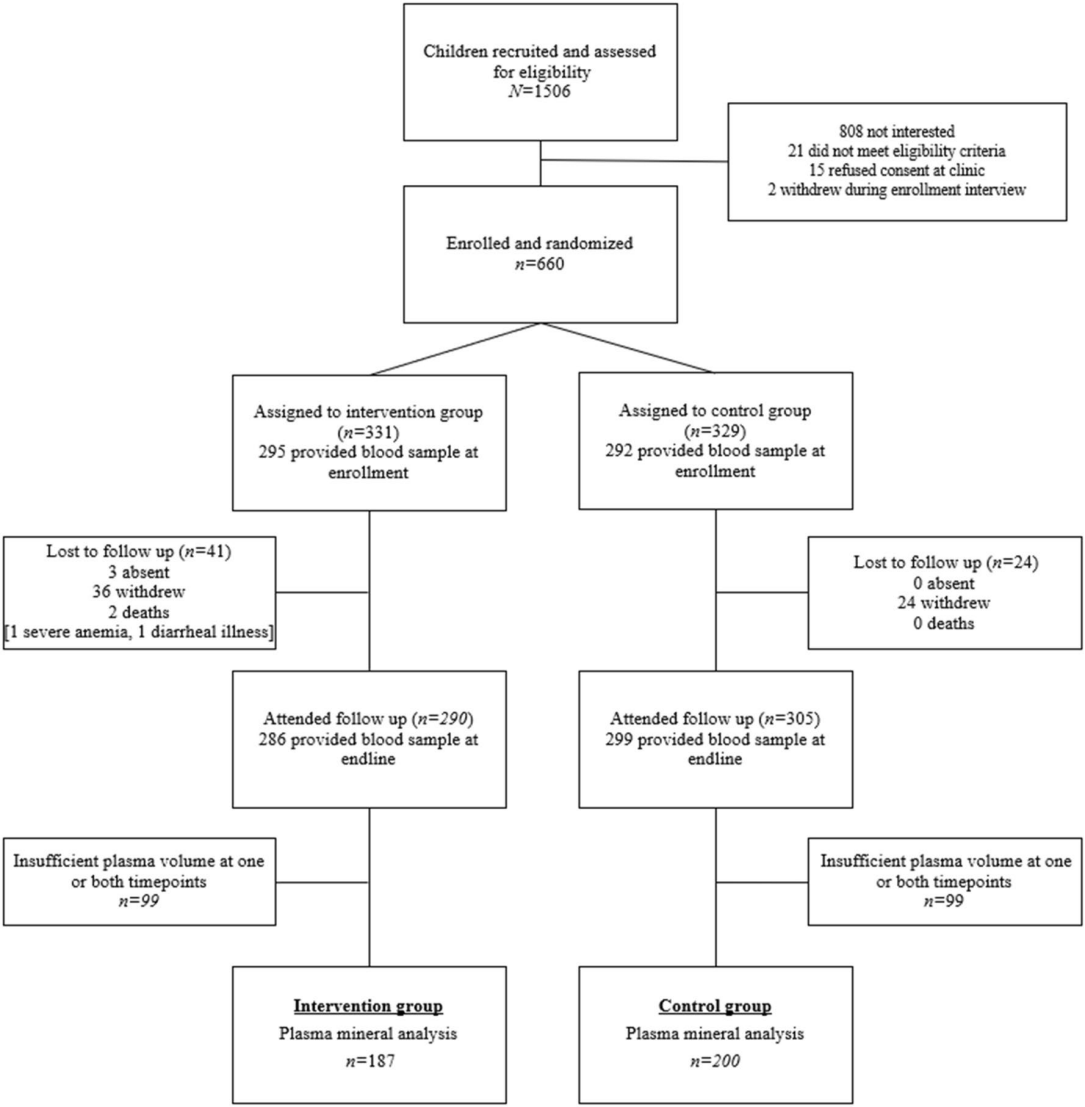
Participant flow diagram for the mineral analysis of the Mazira Project.

This analysis included 187 children in the intervention group and 200 children in the control group who had sufficient plasma samples at both baseline and follow-up (Fig. 1). The primary reasons for withdrawal included familial disagreement on participation (53%), relocation (15%) and child refusal to eat eggs (6%). Baseline characteristics for children with a convenience sample of available plasma were similar between groups with respect to age, sex, diet, and maternal and household characteristics (Table 1). There were few exceptions, including mothers in the intervention group had significantly higher levels of formal education and were more likely to be literate tha n those in the control group. Additionally, a higher percentage of children in the egg group had consumed fish in the day prior to enrollment compared to those in the control group.

**Table 1:** Baseline characteristics for Mazira Project participants included in the mineral analysis, 2018–2019.

Characteristics	Control (n = 200)	Intervention (n = 187)
	% Or mean (SD)	% Or mean (SD)
**Child characteristics**		
Child age (m)	7 (1.2)	7 (1.2)
Female	50	46
Firstborn	25	32
Malaria	12	14
Anemia (< 11 g/dL)	61	64
Breastfeeding	100	100
Consumed dairy in past 24 h	8	9
Consumed meat in past 24 h	2	3
Consumed fish in past 24 h	21	30
Consumed eggs in past 24 h	4	4
**Maternal characteristics**		
Maternal age (y)	26 (6.9)	26 (6.6)
***Maternal education***		
No formal education	22	11
Incomplete primary	62	60
Completed primary or greater	17	29
Maternal literacy	45	53
***Maternal marital status***		
Unmarried	24	29
Monogamous	55	52
Polygamous	22	19
***Maternal occupation***		
Farming or fishing	39	41
Service	24	25
Housewife	37	33
**Household characteristics**		
Number children < 5y	2 (0.8)	2 (0.9)
***Food insecurity category***^1^		
None	15	23
Mild	4	3
Moderate	8	9
Severe	73	65
Distance to water source ≥ 10 min	54	55
Number of rooms in home	3 (1.2)	3 (1.2)
Own latrine	96	97
Poor floor quality^2^	75	74
Poor roof quality^2^	63	57
Poor wall quality^2^	46	43
Any cows owned	2	2
Any goats owned	22	21
Any chickens owned	35	33
**Health center catchment area**		
Lungwena	49	49
Malindi	52	51

^1^Food insecurity assessed using the Household Food Insecurity Access Scale^29^.

^2^Poor qualities defined as straw, grass, mud, or unburnt brick.

Child, maternal and household characteristics of participants included in this analysis and those excluded due to insufficient plasma samples were similar (Supplementary Table S4), except mothers of included participants were more likely to have higher levels of formal education and more likely to be in monogamous relationships. Additionally, there was a higher selection of mother–child dyads living in the Malindi health center catchment area in this analysis, compared to those excluded.

### Plasma mineral distributions and prevalence of deficiency

**Figure 2:**
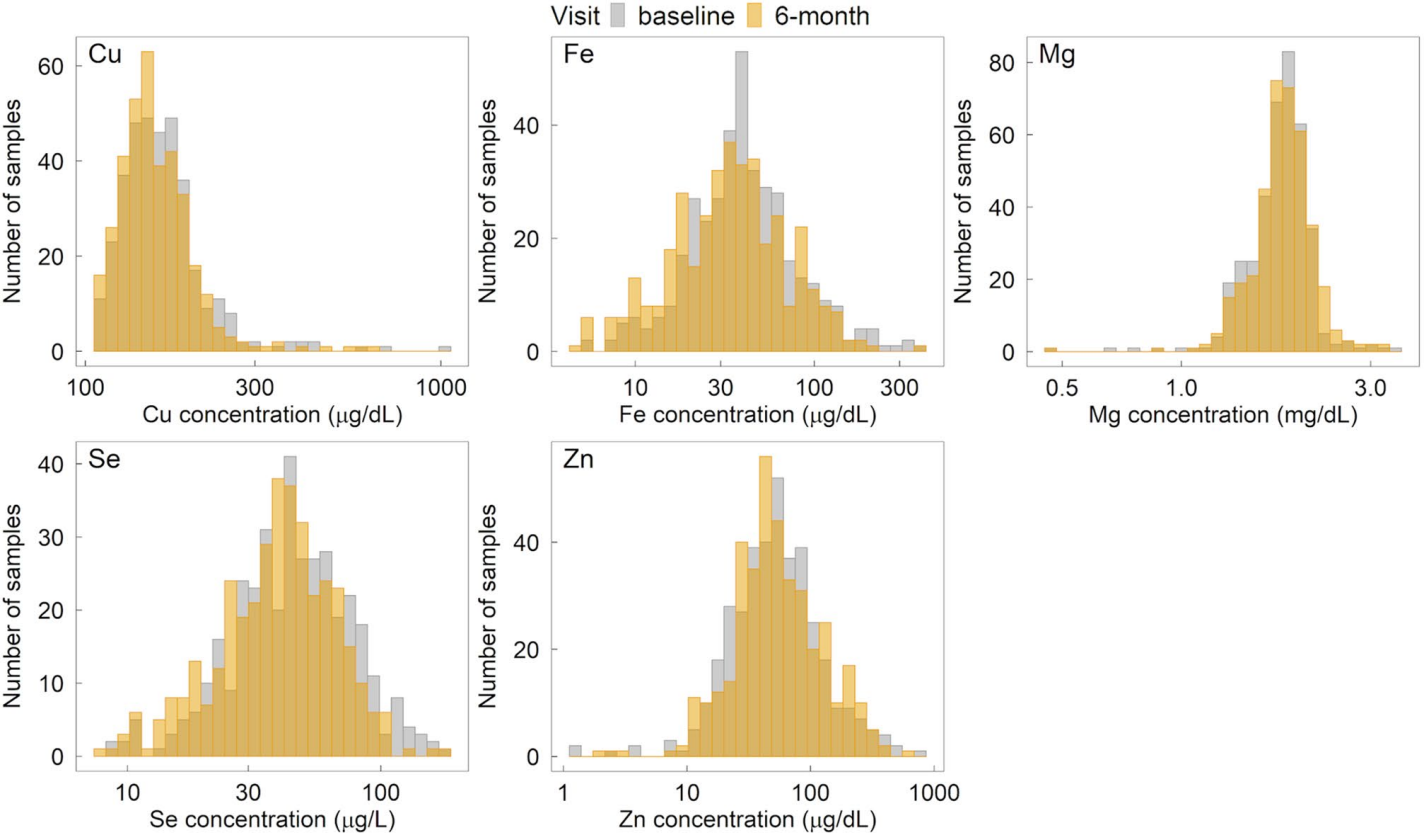
Distribution of plasma mineral concentrations of participants in the Mazira Project, 2018–2019^1^. ^1^Distribution of plasma mineral levels for all participants at baseline and the 6-month follow-up shown by histograms.

The distributions of plasma copper, magnesium, selenium, zinc, and iron concentrations were similar from baseline to follow-up (Fig. 2). Among participants, prevalence of zinc deficiency was 57.4% at baseline and 60.5% at the 6-month follow-up. Severe hypomagnesaemia was seen in 0.5% (n = 4 at baseline and n = 2 at follow-up) of participants. A total of 7% (n = 29 at baseline and n = 25 at follow-up) had plasma copper levels that were below the minimum detectable range (108 µg/dL). The remaining samples showed plasma copper concentrations either within the reference range for age or above. Mean baseline plasma calcium levels, based on the presence of calcium isotope 43, were 4.77 mg/dL (SD 0.53) and 4.86 mg/dL (SD 0.61) for the control and intervention groups, respectively. At follow-up, the mean calcium levels in the control group were 4.67 mg/dL (SD 0.52) and 4.63 mg/dL (SD 0.49) in the intervention group. These findings were not included in the regression models due to concerns about valid estimations of total calcium levels using this methodology.

### Effect of intervention on mineral status

There were no mean differences in plasma mineral concentrations between groups at the follow-up, apart from iron (Table 2). Children in the intervention group had significantly lower plasma iron concentrations in fully adjusted models compared to children in the control group.

**Table 2:** Plasma mineral concentrations and adjusted differences compared at baseline and follow-up between control and intervention group of children in the Mazira Project.

	Baseline		6-month		Minimally adjusted^1^ models	Fully adjusted^2^ models
	Control (n = 200)	Intervention (n = 187)	Control (n = 200)	Intervention (n = 187)		
	Mean (SD)	Mean (SD)	Mean (SD)	Mean (SD)	MD (95% CI)	MD (95% CI)
Plasma magnesium (mg/dL)	1.78 (0.31)	1.79 (0.32)	1.83 (0.32)	1.82 (0.32)	− 0.01 (− 0.08, 0.05)	− 0.01 (− 0.08, 0.05)
Plasma copper (µg/dL)	155.96 (43.20)	175.17 (99.02)	156.09 (57.05)	161.06 (60.80)	3.88 (− 7.95, 15.72)	3.88 (− 7.95, 15.72)
Plasma iron (µg/dL)	51.27 (45.82)	51.00 (49.20)	46.35 (37.15)	37.26 (29.46)	− 9.29 (− 15.95, − 2.64)	− 9.07 (− 15.63, − 2.51)
Plasma selenium (µg/L)	47.41 (28.48)	50.59 (28.58)	42.64 (25.16)	42.94 (21.65)	− 1.03 (− 5.13, 3.08)	− 0.35 (− 4.48, 3.79)
Plasma zinc (µg/dL)	73.16 (74.28)	75.21 (88.23)	74.16 (67.48)	74.64 (75.76)	0.60 (− 13.57, 14.78)	− 0.60 (− 13.57, 14.78)

^1^Minimally adjusted for baseline concentrations, time of sample collection, and time of last meal.

^2^Fully adjusted for age, sex, maternal education, household index, and number of children in the household under five years of age when indicated. CI = confidence interval; MD = mean difference; SD = standard deviation.

### Effect modification of mineral status

Effect modification analyses did not identify any significant modifiers in the relationship between the intervention and plasma mineral levels.

## Discussion

Minerals are necessary for proper cellular function in infants and children, especially during critical periods of growth and development. In this study of young Malawians, the egg intervention did not change plasma magnesium, copper, selenium, or zinc concentrations compared to the control group. At the 6-months, plasma iron concentrations were significantly lower in the intervention group compared the control group. Additionally, the likelihood of mineral deficiency was particularly concerning for zinc.

Plasma selenium levels were not increased by provision of one egg daily for six months in these young Malawian children. Similar results have been reported in adults who consume eggs daily, but little is known about the effect of eggs in young children^39,40^. Adults have higher recommended dietary allowances (RDAs) of selenium; therefore, one egg provides only 36% of the RDA of selenium compared to approximately 77% of the daily requirement for young children^19,20,41^. Previous reports have shown that egg consumption is associated with higher intakes of selenium, which supports dietary intake data seen in our study^21,24^. Even with high selenium concentrations in the provided eggs and adequate intake reports, plasma selenium levels decreased from baseline to follow-up in both groups. Given the age of the children in the study, this contradicts the typical response of circulating selenium levels, as previous studies on healthy infants have shown levels decrease from birth to 4 months of age and then start to increase, with median plasma selenium levels of 49 µg/L between 4–12 months and 71 µg/L between 1–5 years of age^42^. Selenium within the plasma is bound to lipoproteins, and in states of malnutrition, lipoprotein synthesis is reduced. Malnutrition thus, may secondarily result in decreases in plasma selenium content, which is not necessarily reflective of total body selenium status^43–45^. This may explain our findings here, as children in both cohorts had high rates of stunting and underweight status at the 6-month follow-up^25^.

At baseline, prevalence of zinc deficiency was found to be equivalent to previous reports ranging from 60–66% in children and adults living in Malawi^46^. This can be explained in part by the high prevalence of dietary zinc inadequacy at baseline^24^. In the context of the baseline deficiencies found in these children, and the increased physiologic demand for adequate zinc intake during these times of rapid growth, the egg alone provides approximately 43% of the child’s RDA of zinc, which would not be enough to surpass their baseline shortcomings and ameliorate the zinc deficiencies^7,47^. Other important variables to consider are the interactions between minerals, bioactive compounds in eggs, and other components of the complementary feeding diet. These interactions can impact mineral absorption, metabolism, and ultimately the mineral status of young children^48^.

Iron can be particularly sensitive to other components of the food matrix. As shown in adults, iron bioavailability can be decreased by proteins like phosvitin, ovalbumin, and ovotransferrin, which are all found in eggs. These proteins lead to iron chelation and decrease its absorption in processes intended to prevent microbial growth in eggs^49^. A study conducted by Makrides et al. showed adding four egg yolks a week for 6-months into infant diets improved plasma iron levels^50^. This suggests that the egg white may have impeded iron absorption in those in the intervention group. Other factors potentially affecting both the intervention and control group may also contribute to impaired iron absorption. For example, maize is a major component in the diet of Malawian children. Maize is high in phytates, antioxidant compounds which have also been shown to hinder iron absorption^51^. Plasma iron levels also have diurnal variations, and are negatively affected by infection, inflammation, and poor enteric health^6^. The combination of inadequate iron intake, and the introduction of foods that may inhibit its absorption, and a high prevalence of chronic infections in this population may account for the high prevalence of iron and zinc deficiencies in this population^24,26,52,53^. Since plasma iron levels should not be used as the sole method to assess iron status, a separate analysis was previously published demonstrating that ferritin, soluble transferrin receptor concentrations, body iron stores, and hemoglobin were not affected by the intervention in this study^26^. These findings suggest that although eggs may not improve iron status, they will not contribute to iron deficiency anemia.

The Mazira Project was designed as a replication trial building on the Lulun Project in Ecuador. The Lulun trial found significant improvements after intervention on linear growth velocity and plasma DHA and choline levels^14,54^. Another study by Bierut and colleagues found that bovine colostrum/egg intervention reduced stunting and increased plasma choline concentration in young Malawian children^23^. Mineral status was not reported in either of these studies and is rarely reported in other similar trials. More commonly, concentrations of plasma minerals and trace elements were assessed after direct use of supplemental micronutrient powders or ready to use foods to increase zinc and iron levels^55,56^.

One notable difference in this Malawian context compared to previous studies was type of foods reported on dietary intake surveys^24,57^. For example, fish intake was reportedly higher in Malawian children than in Ecuadorian children. However, further inquiry revealed that Malawian infants were given broth made using fish, but very little meat, and were therefore not gaining beneficial calories and nutrients, including iron, calcium, and zinc, provided by fish intake^58^. Children in the Mazira project had overall lower total intakes of protein, vitamin C, iron and zinc from complementary foods compared to the children living in Ecuador^57^. The nutritional gaps of young children living in Malawi may be greater than those living in Ecuador, and the egg intervention was not sufficient to overcome these mineral and nutrient deficits. Additional research is necessary to determine the impacts of egg intervention on mineral status of young children across multiple contexts.

There were strengths and limitations to acknowledge in this study. It was a rigorously designed randomized control trial with high adherence (71%) to the intervention based on 24-h recall at the 6 month follow-up and a comprehensive set of mixed methods including detailed dietary recalls^25^. There was a large sample size for detecting intervention effects on several nutrient biomarkers. One limitation for this analysis was the loss in numbers of samples with sufficient volume, although groups remained comparable across characteristics. Other assays conducted previously to look at nutrient biomarkers required more blood than anticipated. Furthermore , venous samples were obtained on non-fasting infants and times ranged throughout the day. This may result in greater variability but was adjusted for during statistical analysis. Another potential limitation was the inability to measure the concentrations of minerals within a complex of protein carriers, such as, lipoproteins in the case of selenium, or body storages. It is possible that there was an increase in total body mineral content that we were not able to account for with our analysis.

Lastly, we were unable to determine total calcium levels or the effect of the intervention in these Malawian children. We attempted to use ICP-MS to measure calcium isotope 43, which resulted in a good recovery. In general, the abundance of Ca (43) is 0.13%, which was used to extrapolate total calcium concentrations. However, levels were found to be considerably lower than normal ranges for young children^59^. Thus, a determination was made that the method did not yield valid calcium values, If the abundance of Ca (43) differs from previous reports of 0.13%, then the calculated total calcium levels will be inaccurate. Given these findings, alternative measures to determine calcium concentration in this population should be considered.

## Conclusions

Young children living in LMICs are particularly vulnerable to malnutrition, multiple mineral deficiencies, and their compounding detrimental effects. To our knowledge, this is the first study to examine multiple plasma mineral concentrations in infants and young children following the provision of one egg per day. Our results did not show improvement in plasma mineral concentrations in these young Malawian children. Additional research is needed to determine culturally and nutritionally relevant ways to improve mineral status and adequate nutrient intake in this context.

## Supplementary Information

Supplementary Information.

## Data Availability

Data described in this manuscript will be made publicly and freely available, along with the code book without restriction at https://osf.io/vfrg7/#!.
