# Supplementary material for: Plasma mineral status after a six-month intervention providing one egg per day to young Malawian children: a randomized controlled trial
Source: Sci Rep. 2023 Apr 24;13:6698. doi: 10.1038/s41598-023-33114-1 (PMC10125967; doi:10.1038/s41598-023-33114-1)
Supplement: Supplementary file 1 — Supplementary Information. [file 41598_2023_33114_MOESM1_ESM.pdf]

Title: Plasma mineral status after a six-month intervention providing one egg per day to young Malawian children: a randomized controlled trial

## SUPPLEMENTAL INFORMATION

**Supplementary Table S1. Operating conditions and instrument settings used in obtaining mineral concentrations using the ICP-MS (NexION® 2000, Perkin-Elmer)**

| | |
|---|---|
| Nebulizer | Concentric (MEINHARD® Plus Glass CT R+) |
| Spray chamber | Glass cyclonic at 2°C |
| Injector | 2.5 mm Quartz |
| Nebulizer Flow | 1 L/min |
| Plasma Gas Flow | 16 L/min |
| RF Power | 1600 W |
| Dwell Time | 25 ms |
| Scanning Mode | Peak hopping |
| Sweeps/Reading | 20 |
| Readings/Replicate | 1 |
| Number of Replicates | 3 |

**Supplementary Table S2. Analyte minimum detection limit and percent recovery determined for mineral analyses on ICP-MS**

| Analyte | Mode used on ICP-MS | Minimum Detection Limit[1] | Unit | Percent Recovery, Mean (SD) |
|---|---|---|---|---|
| Ca (43) | Standard | 1.90E-01 | mg/dL | 92.5 (7.4) |
| Cu (63) | Collision | 1.08E+02 | µg/dL | 110.2 (6.2) |
| Fe (57) | Collision | 4.83E+00 | µg/dL | 113.4 (6.8) |
| Mg (24) | Collision | 8.23E-03 | mg/dL | 105.6 (5.3) |
| Se (78) | Standard | 7.55E+00 | µg/L | 108.6 (4.0) |
| Zn (66) | Collision | 1.24E-01 | µg/dL | 102.7 (8.3) |

[1] Minimum detection limit calculated from the sum of the average of method blanks ($n = 7$) and the product of the Student's t-value approximate for the single-tailed 99th percentile t statistic with n-1 degrees of freedom and the sample deviation [MDL=x ̅+t_ ((n-1,1- ∝=0.99))*σ)]; method from 40 C.F.R. § 136.

**Supplementary Table S3. Reference ranges and cutoffs used to determine prevalence of mineral deficiencies**

| Mineral | Units | Specimen type[2] | Lower limit | Upper limit | Cutoff for prevalence of deficiency[3] |
|---|---|---|---|---|---|
| Copper | µg/dL | serum | 90 | 190 | < 50 |
| Iron | µg/dL | serum | 16 | 128 | |
| Magnesium | mg/dL | serum | 1.7 | 2.1 | < 1 (severe) |
| Selenium | µg/L | serum | 16 | 71 | |
| Zinc | µg/dL | plasma | 80 | 120 | < 65 (morning/non-fasting)<br>< 57 (afternoon) |

[1]Reference ranges are presented based on those seen in healthy children and adults [34]
[2]Indicates type of specimen used for reference. Plasma and serum levels do not significantly differ in concentrations of magnesium, zinc, and selenium. Plasma copper levels may be slightly higher than serum measurement, although difference is minimal [59].
[3]Thresholds for zinc are based on previously accepted guidelines, and were determined based on time of blood draw for each participant [7].

**Supplementary Table S4. Characteristics compared between those included in mineral analysis and those excluded**

| Characteristics | Included in analysis (n = 387) | Excluded from analysis (n = 275) | |
|---|---|---|---|
| | % Or Mean (SD) | % Or Mean (SD) | *p* |
| **Child characteristics** | | | |
| Child age (m) | 7 (1.2) | 7 (1.2) | 0.455 |
| Female | 48 | 49 | 0.746 |
| Firstborn | 28 | 27 | 0.843 |
| Malaria | 13 | 11 | 0.535 |
| Anemia | 62 | 58 | 0.256 |
| Breastfeeding | 100 | 100 | 0.184 |
| Consumed dairy | 8 | 9 | 0.821 |
| Consumed meat | 3 | 1 | 0.063 |
| Consumed fish | 25 | 30 | 0.117 |
| Consumed eggs | 4 | 5 | 0.472 |
| **Maternal characteristics** | | | |
| Maternal age (y) | 26 (6.7) | 26 (6.8) | 0.635 |
| ***Maternal education*** | | | |
| No formal education | 16 | 21 | 0.04* |
| Incomplete primary | 61 | 64 | |
| Completed primary or greater | 23 | 16 | |
| Mother can read | 49 | 42 | 0.066 |
| ***Maternal marital status*** | | | |
| Unmarried | 27 | 16 | 0.003* |
| Monogamous | 53 | 64 | |
| Polygamous | 20 | 20 | |
| ***Maternal occupation*** | | | |
| Farming or fishing | 40 | 48 | 0.161 |
| Service | 25 | 21 | |
| Housewife | 35 | 31 | |
| **Household characteristics** | | | |
| Number of children under 5y | 2 (0.8) | 2 (0.8) | 0.397 |
| ***Food insecurity category***[1] | | | |
| None | 19 | 19 | 0.764 |
| Mild | 4 | 2 | |
| Moderate | 9 | 9 | |
| Severe | 69 | 70 | |
| Walking distance to water source ≥10 min | 46 | 43 | |
| Number of rooms in home | 3 (1.2) | 3 (1.3) | 0.661 |
| Own latrine | 96 | 97 | 0.921 |
| Poor floor quality[2] | 75 | 79 | 0.209 |
| Poor roof quality[2] | 60 | 62 | 0.654 |
| Poor wall quality[2] | 44 | 43 | 0.852 |
| Any cows owned | 2 | 4 | 0.113 |
| Any goats owned | 21 | 15 | 0.048* |
| Any chickens owned | 34 | 31 | 0.426 |
| **Health center catchment area** | | | |
| Lungwena | 49 | 60 | 0.006* |
| Malindi | 51 | 40 | |

*P<0.05

[1] Food insecurity assessed using the Household Food Insecurity Access Scale [29]

[2] Poor qualities defined as straw, grass, mud or unburnt brick.
